# 

**Published:** 1892-05

**Authors:** 


### AMERICAN JOURNAL

OF

### DENTAL SCIENCE,

EDITED BY

F. J. S. GORGAS, M. D., D. D. S.

RICHARD GRADY, M. D., D. D. S

VOLUME XXVI—Third Series.

D 220

BALTIMORE:
SNOWDEN & COWMAN.

LONDON:
TRUBNER & CO., 60 PATERNOSTER ROW.
1893.

### Index to Vol. XXVI.—Third Series.

Annual Meeting of the Chicago Dental Society, - 41
A Difference of Opinion, - - - - - 48
Articulation of the Teeth, - - - - 72
Acute Coryza, - - - - - 95
Aristol, - - - - - - - 138
Arrest and Trial of a Traveling Medical Man, - 139
A Practical Cheoplastic Plate, - - - - 149
Amalgam Filling, - - - - - 178
Anæsthesia and Anæsthetics, - - - - 193
A Plea for the Preservation of the Natural Teeth, - 210
Amalgams, - - - - - - 237
Avoid Lengthy Operations for Children, - - 238
A Nickel Removing Solution, - - - - 240
An Abnormal Pulp Canal, - - - 273
A New Regulating Device, - - - - 277
A Fatal Curiosity, - - - - - 335
A Process for Soldering Glass and Porcelain with Metals, 336
A Case of Pyorrhœa Alveolaris, - - - 337
Application of the Rubber Dam, - - - 345
Arrogant Critics, - - - - - 378
Artificial India Rubber, - - - - - 379
Another Case of Insanity Caused by Nitrous Oxide Gas, 379
An Infant Born with Teeth, - - - - 423
Amœbæ in an Abscess of the Jaw, - - - 424
Ancient Jewel-pointed Tools, - - - - 425
An Over-production of Scholars, - - - 426
A Woman Swallows a Razor, - - - - 426
A Fatal Case of Noma in an Adult, - - 427
A Treatise on Nervous and Mental Diseases, - - 428
Anchorage of Gold Fillings, - - - 440
A Few Hints to Students, - - - - 457
Application of the Rubber Dam, - - - 467
A Chance for Every Tooth, - - - - 479

Anchorage of Gold Fillings, - - - 501

Asbestos Fiber in Root-Canal Treatment, - - 506

A Study of the Diseases of the Peridental Membrane, having their Origin at or near the Gingival Margin, 508

Abscessed Teeth, - - - - - 542

A New Lining for Vulcanite Plates, - - - 564

A Demonstration of Hypnotism, - - - 565

A Man who is Fed Through a Funnel, - - 572

A Series of Questions and Answers for Dental Students, 573

A Temporary Crown, - - - - 576

Baltimore Dentists in Germany, - - - 231

Clear Shellac Varnish, - - - - 96

Causes of Pain Referred to the Teeth, - - 124

Courts Differ in Regard to Inhaling Gas, - - 140

Copper Amalgam, some of its Mechanical Uses, - 155

Constructing Crown-and-Bridge-work, - - 219

Combination Fillings, - - - - 228

Compound Fillings, - - - - - 236

Combination of Cement with Amalgam - - 238

Culture and Professional Success, - - - 281

Chicago Dental Society, - - - - 289

Cocaine, - - - - - - 332

Clean Instruments, - - - - 335

Cold in the Head, - - - - - 335

Copper Amalgam—Discrimination in Its Use Necessary to Success, - - - - - 355

Caries, - - - - - - - 417

Cohesive Gold for Filling, - - - - 449

Chapin A. Harris, M. D., D. D. S., - - - 529

Cure of Necrosis in the Lower Maxilla, - - 553

Committee on Exhibits World's Columbian Dental Congress, - - - - - - 571

Death in the Operating Chair, - - - 41

Dentists and the Census, - - - - 87

District of Columbia Dental Law, - - - 135

Devitalizing Pulps, - - - - - 144

Dr. W. H. Dwinelle, - - - - 276

Disinfection of the Hands, - - - - 287

Dental Law of the District of Columbia, - - 313

Dangerous Instruments. - - - - - 335
Dental Erosion and the Gouty Diathesis, - 352
Discussion of "Tobacco and Its Effects," - - 360
Discussion upon the Desirability of Extraction of the Sixth-Year Molar, - - - - - 368
Death in a Dentist's Chair, - - - - 380
Death from Syncope, - - - - - 381
Drying of Paints, - - - - - 432
Death from Alveolar Abscess, - - - 527
Dental Commencement of Meharry Medical College, - 571
English Advertising Dentists, - - - 43
Experiments with Pental, - - - - 85
E. Parmly Brown's Porcelain Bridge-and-Crown Work, 120
Ether Versus Chloroform, - - - - 285
Fitting a Cap or a Band to a Root, - - - 76
For the Teeth, - - - - - 143
Failure in Crown Work, - - - - 431
Filling the Roots of Second Molars, - - 436
First and Second Molars, - - - - 528
Gold Alloys, - - - - - - 48
Gold Crowns, - - - - - - 141
Gold for Filling Permanent Teeth, - - 240
Gum Lancing an Important Therapeutical Measure, - 329
Gold Waste of Value, - - - - - 377
Gold Attachments in Cases of Close Bite. - - 525
How to Procure an Impression of the Mouth when Patient is Inclined to Nausea and Vomiting, - 47
Hemorrhage after Extraction. - - - 206
How to Sleep, - - - - - - 576
Injuries to the Gum Septum, - - - 152
Influence of Heredity, - - - - - 283
Illinois State Dental Society and Iowa State Dental Society—Joint Meeting, - - - 573
Lysol, - - - - - - - 480
Maryland State Dental Association, - - 1
Maryland State Dental Association, - - - 40
Melted Gold Fillings, - - - - - 42
Maryland State Dental Association, - - - 49
Medicine in Vegetables, - - - - 93

Manipulating Cements, - - - - - - 94
Maryland State Dental Association, - - 97
Missouri State Dental Association, - - - 191
Mal-Occlusion of Artificial Molar Teeth, - - 284
Microbes, and what they are doing, - - - 303
Micro-Organisms of the Mouth, - - - 385
Medicine as a Career, - - - - - 470
Mechanical Abrasion of the Teeth, - - 493
Menstruation and the Teeth, - - - - 575
National Association of Dental Faculties, - 186
Neuralgia, - - - - - - 192
National Association of Dental Examiners, - 232
Normal Teeth in Children, - - - - 239
Necklace of Teeth, - - - - 328
New Method of Artificial Respiration, - - 330
Opposition to a New Dental College, - - 19
Oral Hygiene, - - - - - 61
Operative Dentistry, - - - - 315
Osseous Union of Temporary Teeth, - - 348
Operation on the Superior Maxilla, - - 423
Ohio State Dental Society, - - - - 477
Operations for Recurring Dislocation of the Lower Jaw, - - - - - 481
On Public Disinfecting Stations and the Use of Steam Under Pressure as a Disinfectant, - - 484
Prosecutions, - - - - - 46
Poison of the Teeth, - - - - 96
Plain Talk on Failures, - - - 145
Pyorrhœa Alveolaris, - - - - 170
Plaster of Paris Formula, - - - 239
Proceedings of the North Carolina Dental Society, 241
Producing Contour Fillings, - - - 299
Professional Dignity, - - - - 384
Production of Platina in Russia, - - - 430
Pyrozone, - - - - - - 498
Points in Extraction, - - - - 537
Root Canals, - - - - - - 131
Retrospect and Prospect, - - - - 376

Root Canal Cleaner, - - - - 528
Restoring the Bite with Gold Crowns, - - - 556
Sinus from an Abscessed Tooth, - - 45
Surgery of the Antrum, - - - - 174
Stove Blacking for Vulcanizer Joints, - - 239
Simple Tests for Impurities in Water, - - 331
Salophein, - - - - - - 334
Sterilizing Sponge in Pulp Capping, - - 350
Sudden Death, - - - - 383
Swallowed a Tooth, - - - 425
Some Properties of Gold Filling, - - - 433
Should Preachers Pay, - - - - 524
Treating a Diseased Antrum, - - - 29
The World's Columbian Exposition, - - 41
The Relative Values of Non-Cohesive and Cohesive Gold, - - - - - - 68
The Noisy Ones, - - - - - 83
To the Dental Profession, - - - - 93
The Way to Raise a Toothache, - - 94
The Contraction of Rubber Plates, - - - 95
The Newest Disinfectants for Root Canals, - 137
The Medical Law as it Concerns Doctors now Practicing, - - - - - - 138
The Odontoblasts, - - - - - 143
The Devitalization of the Dental Pulp, - - 166
The Physiognomical Significance of the Face and Teeth, - - - - - 181
The Dentist a Teacher, - - - - 184
To Clarify Wax, - - - - - 238
The Use of Tobacco, - - - - 240
The Condition of Dentine in Pulpless Teeth, - 266
The Surgery of the Tongue, - - - 279
The Constant Extraction of Permanent Teeth, - 288
The Keeley Cure, - - - - - 333
The First Molar, - - - - 341
The World's Congress Auxiliary of The World's Columbian Exposition. - - - 373

Treating an Exposed, or Partly Exposed, Pulp, 382

The Treatment of the Irregularities of the Teeth, - 403

The Judge and the Doctor, - - - 422

The National Medical Dictionary, - - 429

The Lay of the Land, - - - - 432

The Advantages of Nitrate of Silver in Dental Practice, - - - - - - 445

Toothache: Its Diagnosis and Treatment, - 453

The St. Louis Dental Society, - - - 474

The "Herbst Method of Treating Pulps," - - 477

The Color Question in a Dental College, - - 514

The First Pan-American Medical Congress, - 518

The American Medical Congress, - - - 522

The Keeley Cure, - - - - 557

Teeth and Tooth-Ache, - - - - 559

The Maryland Board of Dental Examiners, - 568

The Baltimore College of Dental Surgery, - - 570

Uses of Electricity, - - - - 522

University of Maryland Dental Department, - 569

Very Rare Indeed, - - - - 288

Vulcanizing, - - - - - 329

Valley Tan, - - - - - 334

Various Blow-pipe Flames, - - - - 430

Why Different Results Follow the Same Line of Treatment in Similar Pathological Conditions in the Oral Cavity, - - - - 160

What is Iron Made of? - - - - 478

World's Columbian Commission—Executive Committee on Awards, - - - 516

Water an Anæsthetic, - - - - 526

in the *Dental Cosmos* to the effect that the Association endorsed, among other recommendations. the extraction of the sixth-year molars.'' To avoid such a construction as to the papers read at the ninth session there is printed with the proceedings the resolution adopted December 6, 1889; to wit, "This Association is not responsible for the views of the authors of the papers published."

---

### ANNUAL MEETING OF THE CHICAGO DENTAL SOCIETY.

THE WORLD'S COLUMBIAN EXPOSITION.—Send fifty cents to Bond & Co., 576 Rookery, Chicago, and you will receive, post paid, a four hundred page advance Guide to the Exposition, with elegant Engravings of the Grounds and Buildings, Portraits of its leading spirits, and a Map of the City of Chicago; all of the Rules governing the Exposition and Exhibition, and all information which can be given out in advance of its opening. Also, other Engravings and printed information will be sent you as published. It will be a very valuable Book and every person should secure a copy.

